# Draft Genome Sequence of Raoultella ornithinolytica P079F W, Isolated from the Feces of a Preterm Infant

**DOI:** 10.1128/MRA.00493-19

**Published:** 2019-08-15

**Authors:** Yuhao Chen, Thomas C. Brook, Cristina Alcon-Giner, Paul Clarke, Lindsay J. Hall, Lesley Hoyles

**Affiliations:** a Department of Surgery and Cancer, Imperial College London, London, United Kingdom; b Department of Biomedical Sciences, University of Westminster, London, United Kingdom; c Gut Microbes and Health Programme, Quadram Institute Bioscience, Norwich, United Kingdom; d Neonatal Intensive Care Unit, Norfolk and Norwich University Hospital, Norwich, United Kingdom; e Department of Biosciences, Nottingham Trent University, Nottingham, United Kingdom; University of Maryland School of Medicine

## Abstract

Here, we describe the draft genome sequence of Raoultella ornithinolytica P079F W, isolated from the feces of an infant residing in a neonatal intensive care unit during an ongoing study to characterize the neonate gut microbiota. P079F W will be used in studies investigating the role of the microbiome in neonatal infections.

## ANNOUNCEMENT

Raoultella ornithinolytica is an inhabitant of aquatic environments, but it is recognized as an emerging and underreported cause of nosocomial infections (1). While its genomic diversity and prevalence in the preterm neonate gut microbiota are poorly understood, R. ornithinolytica has been associated with bacteremia, urinary tract infection, and early-onset sepsis in infants (2–4).

Here, we report the draft genome of an *R. ornithinolytica* strain, P079F W, isolated from the feces of a 12-day-old male preterm infant who had been delivered by Caesarean section at Norfolk and Norwich University Hospital in the United Kingdom. The fecal sample was collected from the infant under ethical approval obtained from the Ethics Committee of the Faculty of Medical and Health Sciences in the University of East Anglia (Norwich, UK) with informed and written consent obtained from the parents. The protocol for feces collection was laid out by the Norwich Research Park (NRP) Biorepository (Norwich, UK), was in accordance with the terms of the Human Tissue Act 2004, and was approved with license no. 11208 by the Human Tissue Authority. The infant had suspected sepsis against a background of prematurity (gestational age, 30 weeks, days unknown; weight, 1,544 g), premature rupture of membranes, and respiratory distress. Chorioamnionitis and funisitis were confirmed on placental histology. Blood culture and lumbar puncture were unremarkable. The infant had a partial septic screen, and intravenous antibiotics (benzylpenicillin and gentamicin) were commenced during the first week of life. After 5 days of antibiotics, the infant showed no signs of infection.

After storage at −80°C, the fecal sample was diluted 1:10 in buffer and plated onto MacConkey agar no. 3 to isolate lactose-positive (pink) colonies. Strain P079F W was isolated along with strain P079F P (5) from the fecal sample. Both isolates were identified by phenotypic testing as Klebsiella oxytoca [API20E code 524577(3/7)]. DNA was extracted from an overnight culture of P079F W using a phenol-chloroform method described fully by Kiu et al. (6) and sequenced using the 96-plex Illumina HiSeq 2500 platform to generate 1,092,878 125-bp paired-end reads (7). Raw data provided by the sequencing center were checked using FastQC v0.11.4 (https://www.bioinformatics.babraham.ac.uk/projects/fastqc/); no adapter trimming was required, and reads had an average Phred score of >25. MetaPhlAn2.6 (8) was used to identify the closest relative of P079F W, leading to a reference-based assembly (against Raoultella ornithinolytica 2-156-04_S1_C1; Assembly accession no. GCA_000703465) being produced by BugBuilder v1.0.3b1 (default settings for Illumina assembly) (9). The genome of P079F W comprised 5,582,297 bp in 47 contigs (*N*_50_ = 295,345), with a G+C content of 55.6% and 5,194 coding sequences (CDS) and 75 tRNAs (NCBI Prokaryotic Genome Annotation Pipeline [10]). It shared 99.59% average nucleotide identity (OrthoANI [11]) with the genome of *R. ornithinolytica* NBRC 105727^T^ (Assembly accession no. GCA_001598295), confirming its affiliation to this species (12–14). Genome completeness was estimated to be 99.84% using CheckM v1.0.13 (15). The strain was capsule type K27 (https://bigsdb.pasteur.fr/klebsiella/) and encodes several virulence factors (yersiniabactin [iron acquisition]; RcsAB [regulation]; type I and type III fimbriae [adherence]) according to a BLASTN search with the draft genome via VFanalyzer (Klebsiella data set) at http://www.mgc.ac.cn/cgi-bin/VFs/v5/main.cgi (16). The genome encodes homologs (strict Comprehensive Antibiotic Resistance Database matches) of antibiotic-resistant determinants associated with efflux pumps and transporters, namely CRP (Antibiotic Resistance Ontology [ARO]:3000518), *marA* (ARO:3000263), *acrB* (ARO:3000216), *msbA* (ARO:3003950), and PmrF (ARO:3003578; linked to colistin resistance) (17).

### Data availability.

This whole-genome shotgun project has been deposited in DDBJ/ENA/GenBank under the accession no. QFTY00000000. Raw sequence reads have been deposited at DDBJ/EMBL/GenBank under accession no. SRR9048023. The version described in this paper is the first version, QFTY01000000.
